# Molecular Differentiation of the African Yellow Fever Vector *Aedes bromeliae* (*Diptera*: *Culicidae*) from Its Sympatric Non-vector Sister Species, *Aedes lilii*

**DOI:** 10.1371/journal.pntd.0004250

**Published:** 2015-12-07

**Authors:** Kelly Louise Bennett, Yvonne-Marie Linton, Fortunate Shija, Martha Kaddumukasa, Rousseau Djouaka, Gerald Misinzo, Julius Lutwama, Yiau-Min Huang, Luke B. Mitchell, Miriam Richards, Eric Tossou, Catherine Walton

**Affiliations:** 1 Computational Evolutionary Biology Group, Faculty of Life Sciences, University of Manchester, Manchester, United Kingdom; 2 Walter Reed Biosystematics Unit, Smithsonian Institution Museum Support Centre, Suitland, Maryland, United States of America; 3 Walter Reed Army Institute of Research, Silver Spring, Maryland, United States of America; 4 Uniformed Services University of Health Sciences, Bethesda, Maryland, United States of America; 5 Department of Entomology, National Museum of Natural History, Smithsonian Institution, Washington, DC, United States of America; 6 Department of Veterinary Microbiology and Parasitology, Sokoine University of Agriculture, Morogoro, Tanzania; 7 Department of Arbovirology, Emerging and Re-emerging Infections, Uganda Virus Research Institute, Entebbe, Uganda; 8 Agro-Eco-Health Platform for West and Central Africa, International Institute for Tropical Agriculture, Republic of Benin; United States Army Medical Research Institute of Infectious Diseases, UNITED STATES

## Abstract

**Introduction:**

Yellow fever continues to be a problem in sub-Saharan Africa with repeated epidemics occurring. The mosquito *Aedes bromeliae* is a major vector of yellow fever, but it cannot be readily differentiated from its non-vector zoophilic sister species *Ae*. *lilii* using morphological characters. Genetic differences have been reported between anthropophilic *Ae*. *bromeliae* and zoophilic *Ae*. *lilii* and between forest and domestic populations. However, due to the application of different molecular markers and non-overlapping populations employed in previous studies, interpretation of species delimitation is unclear.

**Methodology/Principle Findings:**

DNA sequences were generated from specimens of *Ae*. *simpsoni* s.l. from the Republic of Benin, Tanzania and Uganda for two nuclear genes apolipophorin 2 (*apoLp2*) and cytochrome p450 (*CYPJ92*), the ribosomal internal transcribed spacer region (*ITS*) and the mitochondrial cytochrome c oxidase (*COI*) barcoding region. Nuclear genes *apoLp2* and *CYPJ92* were unable to differentiate between species *Ae*. *bromeliae* and *Ae*. *lilii* due to ancestral lineage sorting, while *ITS* sequence data provided clear topological separation on a phylogeny. The standard *COI* barcoding region was shown to be subject to species introgression and unable to clearly distinguish the two taxa. Here we present a reliable direct PCR-based method for differentiation of the vector species *Ae*. *bromeliae* from its isomorphic, sympatric and non-biomedically important sister taxon, *Ae*. *lilii*, based on the *ITS* region. Using molecular species verification, we describe novel immature habitats for *Ae*. *lilii* and report both sympatric and allopatric populations. Whereas only *Ae*. *lilii* is found in the Republic of Benin and only *Ae*. *bromeliae* in Tanzania, both species are sympatric in Uganda.

**Conclusions/Significance:**

Our accurate identification method will allow informed distribution and detailed ecological studies that will facilitate assessment of arboviral disease risk and development of future targeted vector control.

## Author Summary

In Africa, epidemic outbreaks of yellow fever continue despite the availability of an effective vaccine. Effective understanding of disease epidemiology and control requires the ability to reliably identify vectors of yellow fever. The mosquito *Ae*. *bromeliae*, a competent vector of yellow fever virus, cannot be reliably morphologically differentiated from its sister species *Ae*. *lilii*, which does not bite humans and so does not transmit yellow fever. DNA sequencing of four molecular markers allowed comparisons of how they perform at distinguishing these species. We found that the mitochondrial cytochrome c oxidase (*COI*) barcoding region and nuclear apolipophorin 2 (*apoLp2*) and cytochrome p450 (*CYPJ92*) were unable to reliably distinguish these species. Conversely, genetic variation at the internal transcribed spacer region (*ITS*) was able to confirm the vector *Ae*. *bromeliae* and non-vector *Ae*. *lilii* as distinct species. Based on *ITS* sequence differences, we developed a robust molecular method to identify the vector *Ae*. *bromeliae* from its sister species *Ae*. *lilii*. Consequently, we find that these species use the same larval habitats including banana, cocoyam and *Dracena spp*. in Uganda. Whereas only *Ae*. *lilii* appears to be present in Benin and only *Ae*. *bromeliae* in Tanzania, we confirm that both species occur in Uganda. Reliable species designation will promote more detailed studies of distribution, ecology and vector status essential for disease risk assessment and mosquito control.

## Introduction

Correctly identifying the vector species involved in mosquito-borne disease transmission is fundamental to predicting disease outbreaks, ascertaining general risk to the human population and targeting control efforts. Despite this necessity, the reliable identification of mosquitoes is problematic in many cases, including the medically important *Aedes simpsoni* complex. This complex comprises three known species including *Ae*. *simpsoni*, *Ae*. *lilii* and *Ae*. *bromeliae*. Among these three species, *Ae*. *bromeliae* is an important vector of yellow fever virus (YFV) and potentially other arboviruses [1]. Yellow fever has increased in incidence as a result of urbanisation and changes to public health policy [2, 3]. The burden of yellow fever in Africa is estimated to be 130,000 cases a year, 85,000 of which result in deaths, despite the availability of a vaccine [4]. After mass immunisation campaigns at the beginning of the 20^th^ Century, YFV was successfully reduced in targeted countries. However, YFV outbreaks are causing renewed attention. In West Africa, 13 out of 14 countries known to host YFV now report cases regularly and have experienced epidemics since 2000 [5, 6]. Further concern has arisen over identification of a novel YFV genotype implicated in recent outbreaks including the first reported outbreak in Kenya, East Africa (1992–1993) [7]. Related genotypes have also been reported in Sudan in 2003 and 2005 and more recently in northern Uganda in 2010 [8–10]. Although much attention has been given to understanding yellow fever disease epidemiology [11], relatively little is known about the mosquito vectors.

Ten species are currently known within the wider Simpsoni Group (Huang 2004). Within this group, Theobald [12–14] originally described three species belonging to the Simpsoni Complex (*Ae*. *simpsoni*, *Ae*. *bromeliae* and *Ae*. *lilii*). Despite these separate species designations, many entomologists referred to all *simpsoni*-like mosquitoes within this complex as the nominotypical species, *Ae*. *simpsoni* [15–21]. In Uganda, it was found that some populations of *Ae*.*simpsoni* s.l. were anthropophilic and attracted to human bait, whereas others were not attracted to man despite local abundance [20]. Furthermore, mosquitoes collected from zoophilic and anthropophilic populations showed different feeding preferences in the laboratory with a preference for rodents or humans, respectively [20]. These findings led to a re-examination of mosquito morphology and subsequently Huang [22, 23] provided a full description of the component members of the Simpsoni Complex, reviewed their ecology and drew attention to the incorrect use of former nomenclature. *Aedes simpsoni*, which has only been reported from South Africa and Swaziland, is not implicated in human disease transmission [22, 23]. The anthropophilic yellow fever vector *Ae*. *bromeliae* is widespread on the African continent [23]. In contrast, *Ae*. *lilii*, only previously reported from Sudan, Ethiopia and Uganda, has never been reported biting man and is thus not considered to be involved in disease transmission [22, 23]. Although we know these three species have different distributions, biting behaviour and vectorial abilities, these need to be fully characterised. Understanding ecology and epidemiology requires that species can be distinguished from one another. However, controversy over mosquito taxonomy means that the anthropophilic disease transmitting *Ae*. *bromeliae* cannot be reliably distinguished from its zoophilic sister species *Ae*. *lilii* [24, 25].

Large scale detailed studies of the Simpsoni Complex by Huang [22, 23, 26], reported that *Ae*. *simpsoni* s.s. can be distinguished from *Ae*. *bromeliae* and *Ae*. *lilii* in that it has simple claws on the mid tarsi, as opposed to toothed mid-tarsal claws present in the latter two taxa. *Aedes simpsoni* can also be easily distinguished from conspecifics by its distinct tarsomere scaling pattern [22, 25]. However, Jupp & Kemp [24] reported variation in tarsal claw morphology of *Ae*. *simpsoni* and *Ae*. *bromeliae*, and questioned the reliability of this diagnostic character. In this study, we focus on the more widespread taxa of the complex, *Ae*. *bromeliae* and *Ae*. *lilii*. These sister taxa can sometimes be reliably identified morphologically based on tarsomere banding patterns, but only when this character is exhibited at the extremes of its range as banding patterns overlap between the species [25]. Lutwama & Mukwaya [25] questioned the usefulness of tarsomere banding patterns as a diagnostic character for *Ae*. *bromeliae* and *Ae*. *lilii*. They observed variation in scale ornamentation on an almost continuous scale and found that progeny from the same mother could be identified both as *Ae*. *bromeliae* or *Ae*. *lilii* based on this morphological character.

The unreliability and practicality of using morphological based methods for routine field-based identification in the Simpsoni Complex has led to attempts to delimit species boundaries through molecular methods. In a study of the Simpsoni Complex, excluding the southerly distributed *Ae*. *simpsoni*, Mukwaya *et al*. [27] found that anthropophilic populations from Kenya and Uganda form a distinct genetic clade separate from non-sympatric and non-anthropophilic populations from Uganda and Nigeria based on the non-coding internal transcribed spacer (*ITS*) region of ribosomal DNA. In that study, species were designated according to both blood feeding preference (because *Ae*. *lilii* is zoophilic whereas *Ae*. *bromeliae* are human vectors) and on tarsomere banding patterns which were distinctive for some specimens [22, 23, 27]. Recently, Walter *et al*. [28] inferred the presence of *Ae*. *bromeliae* and *Ae*. *lilii* in sympatry in Rabai, Kenya based upon genetic differences between domestic/peri-domestic and forest populations. However, how these putative species relate to those characterised by Mukwaya *et al*. [27] remains unclear as they used different molecular markers; two nuclear genes, apolipophorin 2 and cytochrome p450 (*CYPJ92*).

A 658 bp region of the cytochrome c oxidase (*COI*) gene has been widely adopted as a DNA barcoding standard for species identification because it shows high utility in discriminating between closely related taxa as well as resolving phylogeographic groups within species [29–31]. However, sole use of mitochondrial DNA to delimit species has been questioned because of the potential for pseudogene development and introgression, which may limit the ability of mtDNA markers to resolve closely related species [32, 33]. Combined analysis of both mitochondrial and nuclear genes can improve phylogenetic resolution since these markers evolve at different rates and so target different levels of the phylogenetic tree [34]. Another barcoding candidate is the nuclear internal transcribed spacer (*ITS*) regions of the ribosomal gene cistron that comprises the 18S, 5.8S, and 28S genes, an external spacer region and two internal spacer regions *ITS1* and *ITS2* [35]. The *ITS* regions evolve at a rapid rate in the absence of functional constraint [36, 37]. Because they are tandemly repeated in the genome, the *ITS* spacer regions are also subject to concerted evolution whereby paralogues are homogenised by genetic exchange [36, 37]. Consequently, paralogues remain genetically similar within species while showing high levels of interspecific divergence [37]. Although not useful in all taxonomic groups, *ITS* can differentiate between sister species in a large number of cases and has been widely used to delimit closely related mosquitoes in *Anopheles* complexes [35, 38–42].

We seek to expand previous work on genetic differentiation of *Ae*. *bromeliae* and *Ae*. *lilii* in the Simpsoni Complex by determining how the putative species identified by Mukwaya *et al*. [27] and Walter *et al*. [28] relate to one another. We achieve this by sequencing the same mosquito samples at previously used molecular markers (*apoLp2*, *CYPJ92* and *ITS*). In addition, we were able to evaluate the utility of various molecular markers, including the *ITS* and *COI* regions, in determining species bounds. We apply these findings to develop a molecular identification method based on variation in the *ITS* region to distinguish the disease vector *Ae*. *bromeliae* from its non-vector sister species *Ae*. *lilii*. Applying this method to mosquitoes from Benin, Uganda and Tanzania enabled us to generate reliable findings of their ecology and distribution.

## Methods

### Sample collection

**Fig 1:**
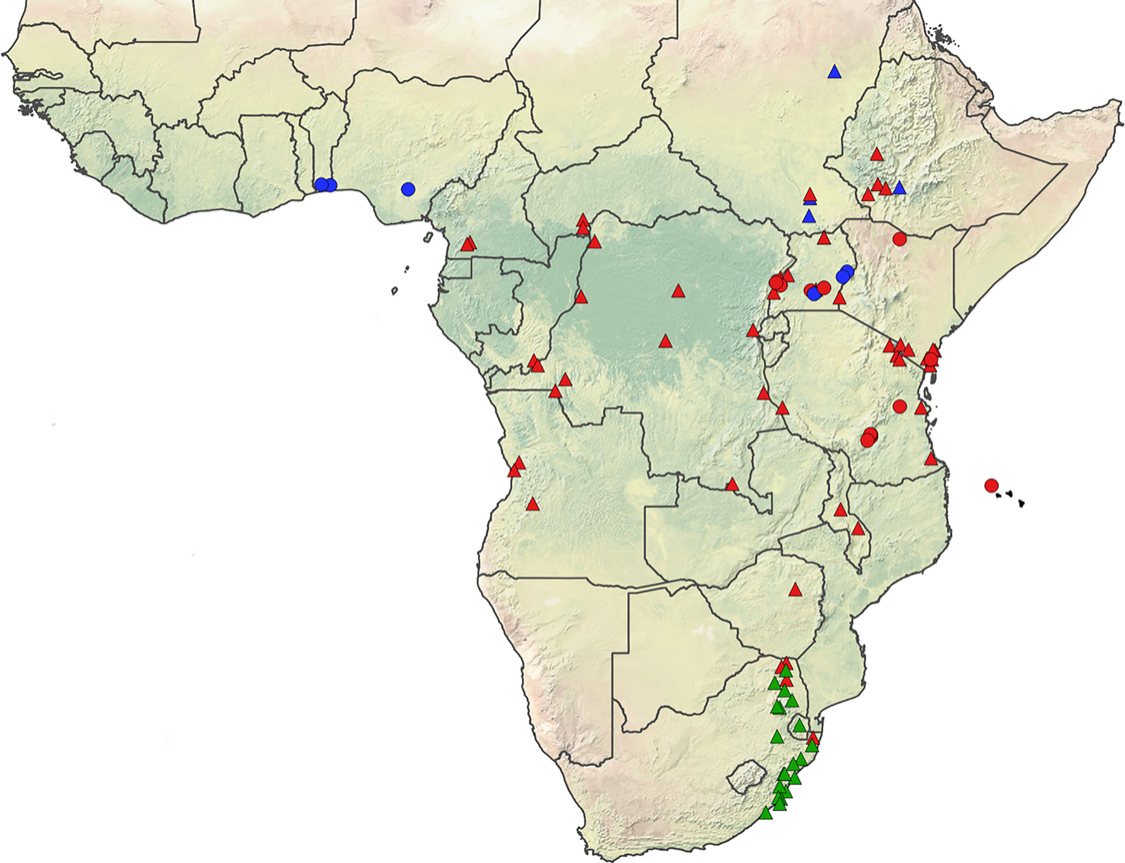
Map of sampling points for *Ae*. *bromeliae* (red circles) and *Ae*. *lilii* (blue circles) used in the current study including samples from Mukwaya *et al*. [27] and Le Goff *et al*. [43]. Also mapped are the previously described sampling points for *Ae*. *bromeliae* (red triangles), *Ae*. *lilii* (blue triangles) and *Ae*. *simpsoni* s.s (green triangles) based on morphological identification, taken from Huang [23]. The map was created in QGIS (QGIS Development Team) made with Natural Earth.

Mosquitoes of the Simpsoni Complex were collected as larvae from natural breeding sites in Tanzania (n = 36), Uganda (n = 50) and the Republic of Benin (n = 24) from locations detailed in Fig 1 and S1 Table from 2009 to 2014. Immature habitats sampled included the leaf axils of *Musa spp*. (banana), *Colocasia spp*. (cocoyam/taro), *Dracaena spp*. and in tree holes. To avoid biasing the dataset with siblings, each discrete habitat was treated as a separate collection and only one individual per collection was taken for genetic analyses. Where possible, immatures were reared though to adults; otherwise, larvae destined for DNA analysis were stored in 95% ethanol. Adults were desiccated with silica for optimal DNA preservation and either stored in BEEM capsules or pinned. Larvae were preserved in ethanol for later extraction of DNA. All adult mosquitoes were identified as belonging to the Simpsoni Complex using the morphological identification key in Huang [26], and a subsample of these, and outgroup taxa including *Ae*. *aegypti* and *Ae*. *aegypti formosus*, were morphologically verified by Dr. Yiau-Min Huang.

### Experimental procedures

DNA was extracted from whole larvae or a single leg of an adult mosquito using the modified phenol-chloroform method in Surendran *et al*. [44]. Forty individuals of the Simpsoni Complex were amplified and sequenced for a region of the mitochondrial *COI* gene with universal primers LCO1490 (5’GGTCAACAAATCATAAAGATATTGG’3) and HCO2198 (5’TAAACTTCAGGGTGACCAAAAAATCA’3) [45] using a protocol recommended by the Consortium for the Barcode of Life (http://barcoding.si.edu/dnabarcoding.htm). Peridomestic *Aedes aegypti* collected from an artificial container in Tanzania was also sequenced at *COI* as an outgroup.

Internal transcribed spacer regions 1 and 2 were amplified from six individuals from Uganda and three individuals from Tanzania that were selected to represent the genetic diversity we observed at the *COI* gene. This was achieved with the 18SFHIN and CP16 primers (5’-GTAAGCTTCCTTTGTACACACCGCCCGT-3’ and 5’-GCGGGTACCATGCTTAAATTTAGGGGGTA-3’, respectively) [46], as used by Mukwaya *et al*. [27]. To generate PCR products, 1 unit of high fidelity MyFi DNA Polymerase (Bioline, UK), 2X MyFi Reaction Buffer, 0.8 μM forward and reverse primer and 1–10 ng of template DNA were used under the following conditions at 30% ramp speed; 95°C for 3 min followed by 30 cycles of 95°C for 30 sec, 58°C for 45 sec and 72°C for 45 sec with no final extension.

Thirteen Individuals, including eight of the same individuals sequenced at the *ITS* region, were also sequenced for regions of the nuclear genes apolipophorin 2 (*apoLp2)* and cytochrome p450 (*CYPJ92)* first described by Brown *et al*. [47] and used by Walter *et al*. [28] in the Simpsoni Complex. This included verified *Aedes aegypti formosus* collected from a tree hole in Tanzania, for use as an outgroup as was the case in Walter *et al*. [28]. Another marker, short-chain dehydrogenase-reductase (*SDR*) also used by Walter *et al*. [28] in the Simpsoni Complex was not used because it produced multiple nonspecific bands on amplification. PCR products were generated as in Walter *et al*. [28]. PCR products were purified with the GenElute PCR clean up kit (Sigma-Aldrich, UK) and Sanger sequenced in forward and reverse directions using the amplification primers. Sequences were generated with BigDye Terminator v3.1 cycle sequencing kit (Applied BioSystems, UK) on an Applied BioSystems 3730 automated sequencer.

*ITS* products were cloned using the P-GEM cloning kit (Promega, UK) as per instructions. Transformants were blue/white screened and colonies with inserts stored in TE buffer for PCR amplification. Universal M13 primers (5’-TGTAAAACGACGGCCAGT-3’ and 5’-CAGGAAACAGCTATGAC-3’) [48] were used to amplify cloned *ITS* products in the following 13μl reaction; 1.25 units of BIOTAQ DNA Polymerase (BioLine, UK), 1 X NH_4_ Reaction Buffer, 2mM MgCl_2_ solution, 0.8mM dNTP and 0.5 μM forward and reverse primer. Thermocycler conditions were 95°C for 2 min followed by 95°C for 15 sec, 60°C for 30 sec and 72°C for 30 sec for 35 cycles and a final extension of 72°C for 10 min. A minimum of two and a maximum of four clones were forward sequenced for each individual.

Cloned *ITS* sequences were aligned with Mukwaya *et al*.’s [27] from several locations in Africa (S1 Table) and Le Goff *et al*.’s [43] sequence data from two *Ae*. *bromeliae* originating from the Indian Ocean island of Mayotte available from GenBank (KF135509-10) using the program Geneious v5.4.7 [49]. Primers were designed based on this alignment to discriminate between species. Several putative species-specific primers were trialled under a wide range of PCR conditions. However, the only primers that worked effectively generated species-specific PCR products that differed by ~30 b.p. Due to this small size difference we recommend that the primer pairs specific for each species are run in separate PCR reactions. Non-specific banding was a feature of all primers tested which we suspect may result from variable indel length among *ITS* copies within an individual. Although PCR protocols were optimised to reduce non-specific banding, some extra banding can be visible but does not obscure amplification/non-amplification of the species-specific PCR product.

The developed primers are nested within the *ITS1-2* region; therefore PCR products encompassing this region were first generated with the 18SFHIN and CP16 primers as described above. These PCR products were purified with the GenElute PCR clean up kit (Sigma, UK) and 0.5 μl was used as template in a 25 μl reaction with 0.6 units of Go Taq Hot Start polymerase (Promega, UK), 1 X NH_4_ reaction buffer, 1 mM MgCl_2_ solution, 0.8 mM dNTP and 0.5 μM forward and reverse primer. Primers developed for amplification of 591 bp in *Ae*. *bromeliae* only were BRO-F (5’-CCTGGCCAGTGGCCA-3’) and BRO-R (5’-GTGCACACCACTGA-3’). Amplification was achieved with a touchdown PCR protocol; initialisation step of 95°C for 3 minutes followed by 95°C for 30 seconds, 82°C for 45 seconds and 72°C for 1 minute for 5 cycles, followed by 30 cycles of 95°C for 30 seconds, 64°C for 45 seconds and 72°C for 1 minute and then a final extension of 72°C for 7 minutes. Primers developed for amplification of a 620 bp region in *Ae*. *lilii* only were LIL-F (5’CTGATGCACTGGCCTCAAAG’3) and LIL-R (5’TCAACCGCCGTGCGTG’3). The thermocycling conditions were 95°C for 3 minutes followed by 95°C for 30 seconds, 78°C for 45 seconds, 72°C for 1 minute for 10 cycles followed by 95°C for 30 seconds, 70°C for 45 seconds, 72°C for 1 minute for 20 cycles and a final extension step at 72°C for 7 minutes. Amplified products were run on a 1.2% agarose electrophoresis gel to determine the presence or absence of DNA bands of the expected size. Positive and negative species controls were used in all PCR reactions. Sequences are available on GenBank (KT998333- KT998452).

### Data analysis

Sequence alignment was achieved with Geneious v5.4.7 [49]. For the *apoLp2* and *CYPJ92* datasets, files were prepared using seqPHASE [50] and PHASE v2.1 was then used to infer haplotypes [51]. The haplotypes of *Ae*. *aegypti formosus* outgroups were determined through alignment with the relevant datasets for *Ae*. *aegypti* in Brown *et al*. [47] and using the program PHASE. Sequences for two *Ae*. *aegypti* from Brown *et al*. [47] were used as outgroups in conjunction with data generated during the present study. The *COI* dataset was aligned with two *COI* sequences of *Ae*. *bromeliae* from the Indian Ocean island of Mayotte available from GenBank (KF135496-97) [43].

Neighbour joining (NJ) trees were constructed in MEGA 6 [52]as in Mukwaya *et al*.[27] and Walter *et al*. [28] using the best available substitution model as chosen by JModelTest [53, 54]. The Tamura-Nei model with uniform rates among sites was used to construct NJ trees for *COI*. The Kimura 2 parameter model (K80) with uniform rates among sites was used for *ITS* sequences and K80 with 0.8 gamma-distributed sites was used to construct trees for *apoLp2* and *CYPJ92*. For all genetic markers, missing data including indels were excluded from analysis. Topological support was determined through 1000 bootstrap replications. The *ITS* sequence tree was constructed without an outgroup because the high level of divergence between the outgroup and the ingroups presents a challenge for sequence alignment. The two *ITS* sequences of *Ae*. *bromeliae* from Mayotte [43] were not included in the NJ tree because they did not overlap with the sequences generated here.

A hierarchical AMOVA was performed on the *ITS* sequence data generated in this study together with Mukwaya *et al*.’s sequences [27] (n = 69) in Arlequin v3.5 [55]. A hierarchical AMOVA was also performed on the *apoLp2* (n = 13) and *CYPJ92* (n = 13) sequences generated in this study.

## Results

### Phylogenetic analysis

**Fig 2:**
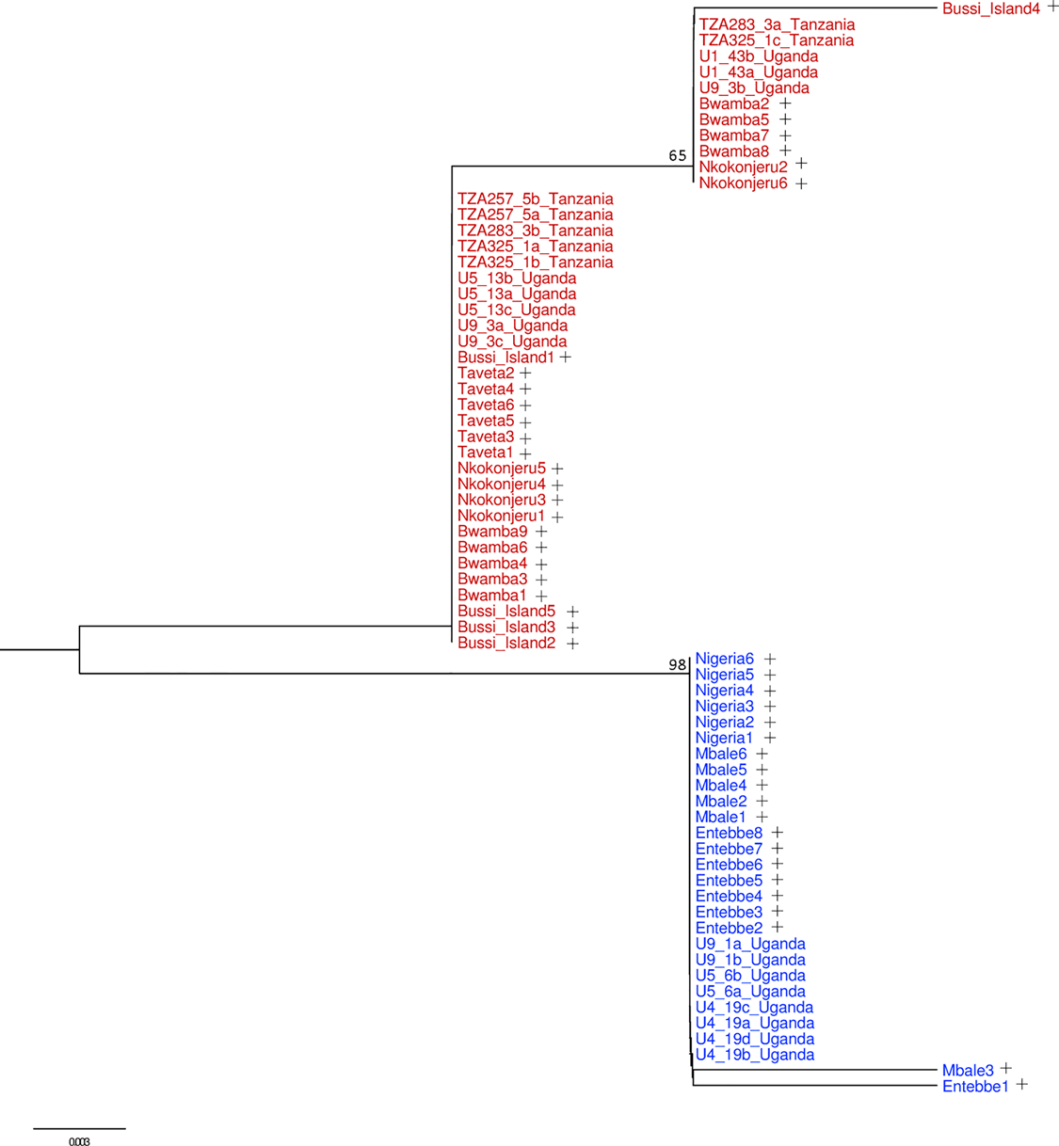
Neighbour joining tree of *ITS* sequence data with labels coloured according to species designation based on *ITS* as in Mukwaya *et al*. [27]. Red labels represent *Ae*. *bromeliae* while blue labels are *Ae*. *lilii*. Sequences from Mukwaya *et al*. [27] (+) are annotated. Bootstrap support values above 55% are shown.

Four fixed point substitutions and eight indels of varying length were observed between species in the first 300 bp of the *ITS* sequence alignment. The neighbour joining tree of *ITS* sequences revealed two major clades with a bootstrap support of 98% (Fig 2). As reported previously, the 46 sequences from Mukwaya *et al*. [27] clustered into one or other of the two clades depending on their inferred host feeding preference and which they accordingly designated as *Ae*. *bromeliae* (anthropophilic) and *Ae*. *lilii* (non-anthropophilic). Sequences of all nine Simpsoni Complex individuals from this study also belonged to one or other of these clades, and we therefore identified them as *Ae*. *bromeliae* or *Ae*. *lilii* with reference to Mukwaya *et al*.’s [27] *ITS*-based species designation.

Mosquitoes collected from Kanyawara, Bundibugyo (Bwamba region) and Najjembe in Uganda represented both *ITS* clades. In comparison, individuals collected from Tanzania strictly aggregated into the *ITS* lineage of *Ae*. *bromeliae*. AMOVA revealed that almost all sequence differences can be explained by variation between species (92.65%), while there is little variation between populations within groups and within populations (0.25% and 7.19%, respectively).

**Fig 3:**
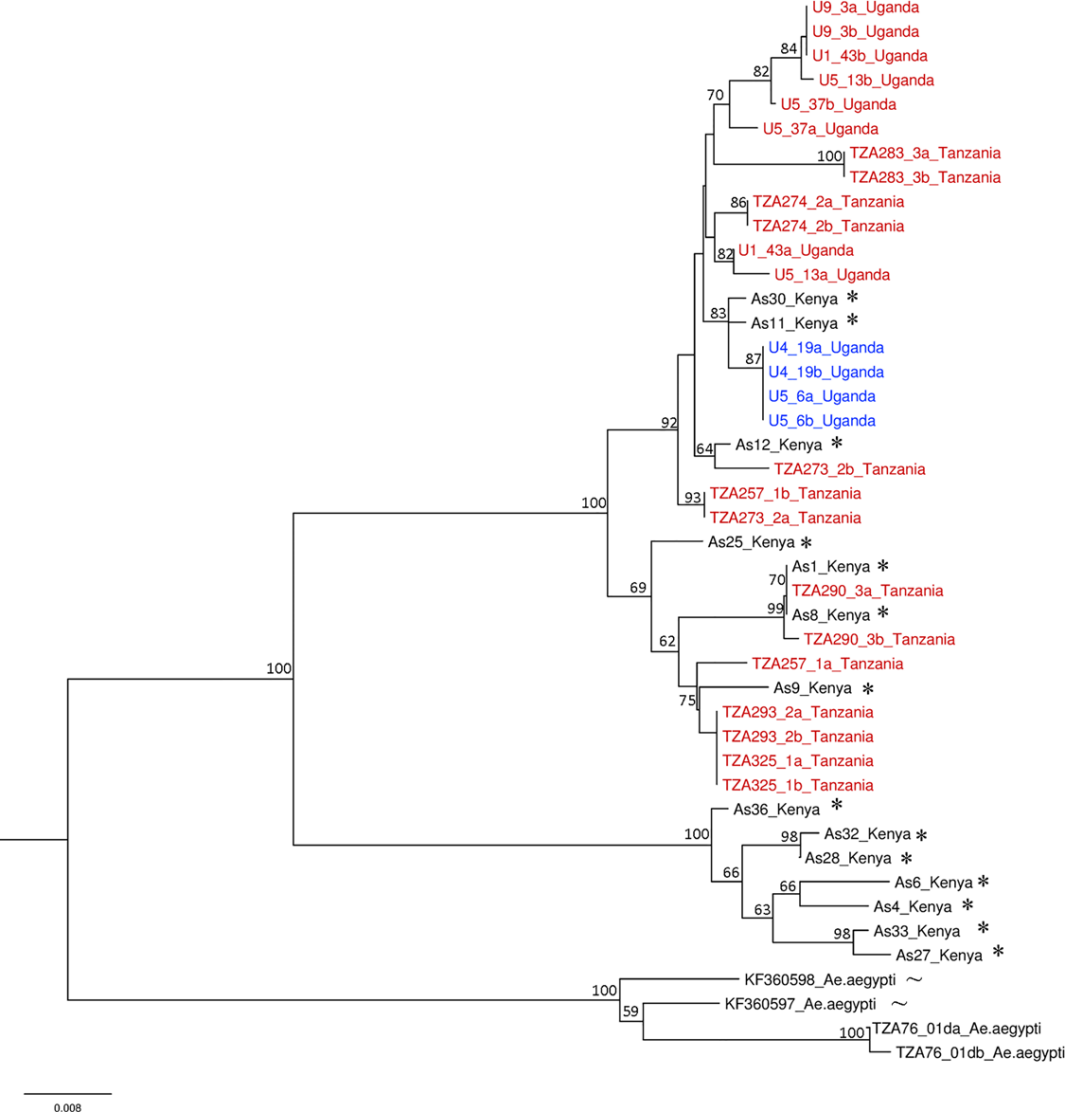
Neighbour joining tree of *apoLp2* sequence data with labels coloured according to *ITS*-based species designation. Red labels represent *Ae*. *bromeliae* while blue labels are *Ae*. *lilii*. Sequences from Walter *et al*. [28] (*) and *Ae*. *aegypti* outgroups (~) from Brown *et al*. [47] are annotated. Bootstrap support values for branches above 55% are shown.

**Fig 4:**
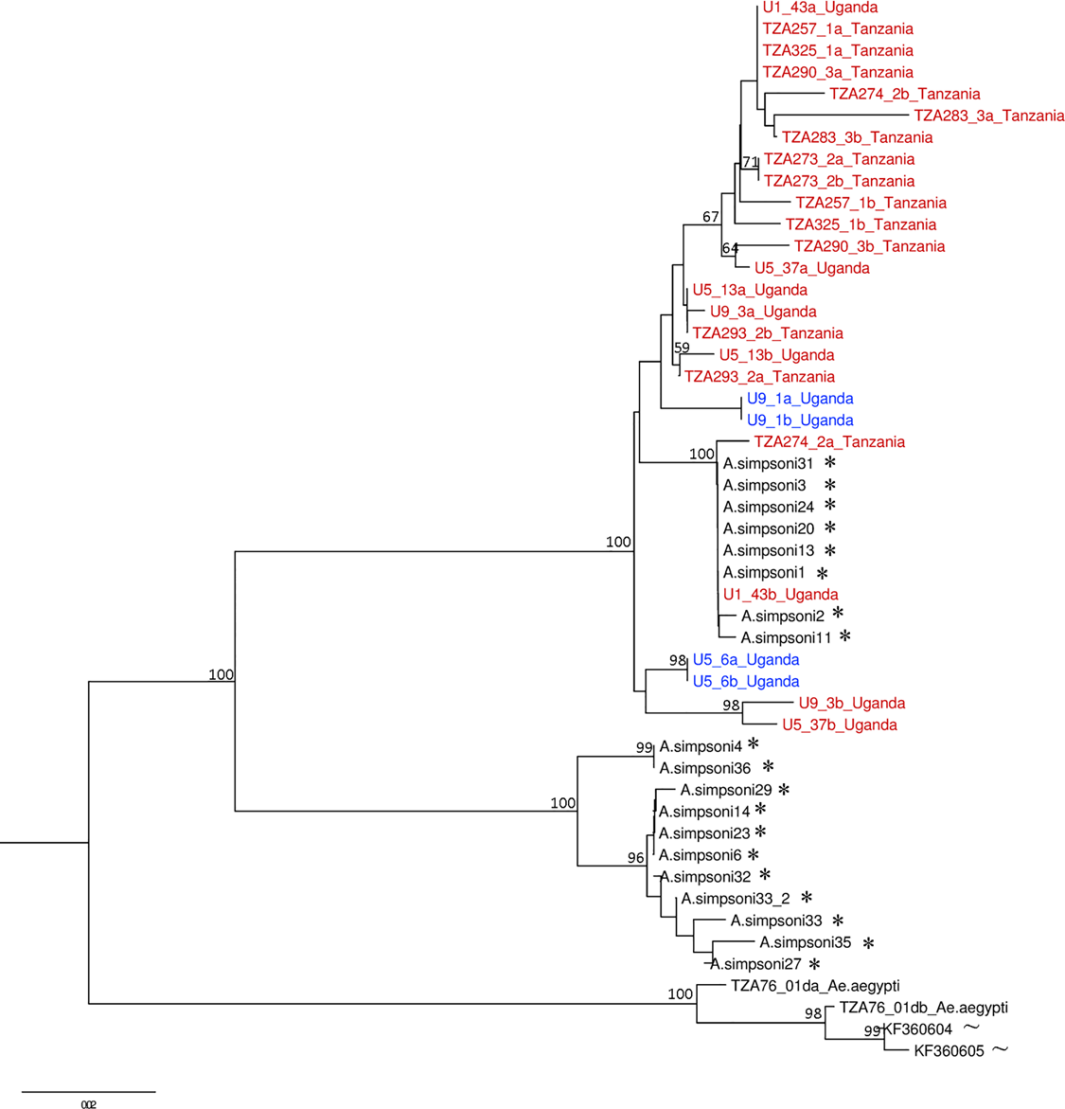
Neighbour joining tree of *CYPJ92* sequence data with labels coloured according to *ITS*-based species designation. Red labels represent *Ae*. *bromeliae* while blue labels are *Ae*. *lilii*. Sequences from Walter *et al*. [28] (*) and *Ae*. *aegypti* outgroups (~) from Brown *et al*. [47] are annotated. Bootstrap support values for branches above 55% are shown.

Sequencing of individuals at both *ITS* and nuclear genes *apoLp2* and *CYPJ92* allowed us to compare the *ITS*-based species designation of Mukwaya *et al*. [27] with that of Walter *et al*. [28]. Two well-supported lineages were observed in NJ trees for the nuclear genes *apoLp2* and *CYPJ92* with 100% bootstrap support (Figs 3 and 4). Individuals confirmed as *Ae*. *bromeliae* or *Ae*. *lilii* using the *ITS* region with reference to Mukwaya *et al*. [27], all fall into a single clade for each of the nuclear genes, *apoLp2* and *CYPJ92*. For both gene trees, the other lineage comprises only the sequences from the forest species in Walter *et al*. [28] that they referred to as *Ae*. *lilii*. A hierarchical AMOVA revealed that nuclear genes *apoLp2* and *CYPJ92* could not effectively distinguish between *Ae*. *bromeliae* and *Ae*. *lilii* with only 0.86% and 18.94% of genetic variance between species groups, respectively. Conversely, there was relatively high genetic variation among populations within species groups (37.04%, 10.28%, respectively) and within populations (62.10%, 70.79%, respectively).

**Fig 5:**
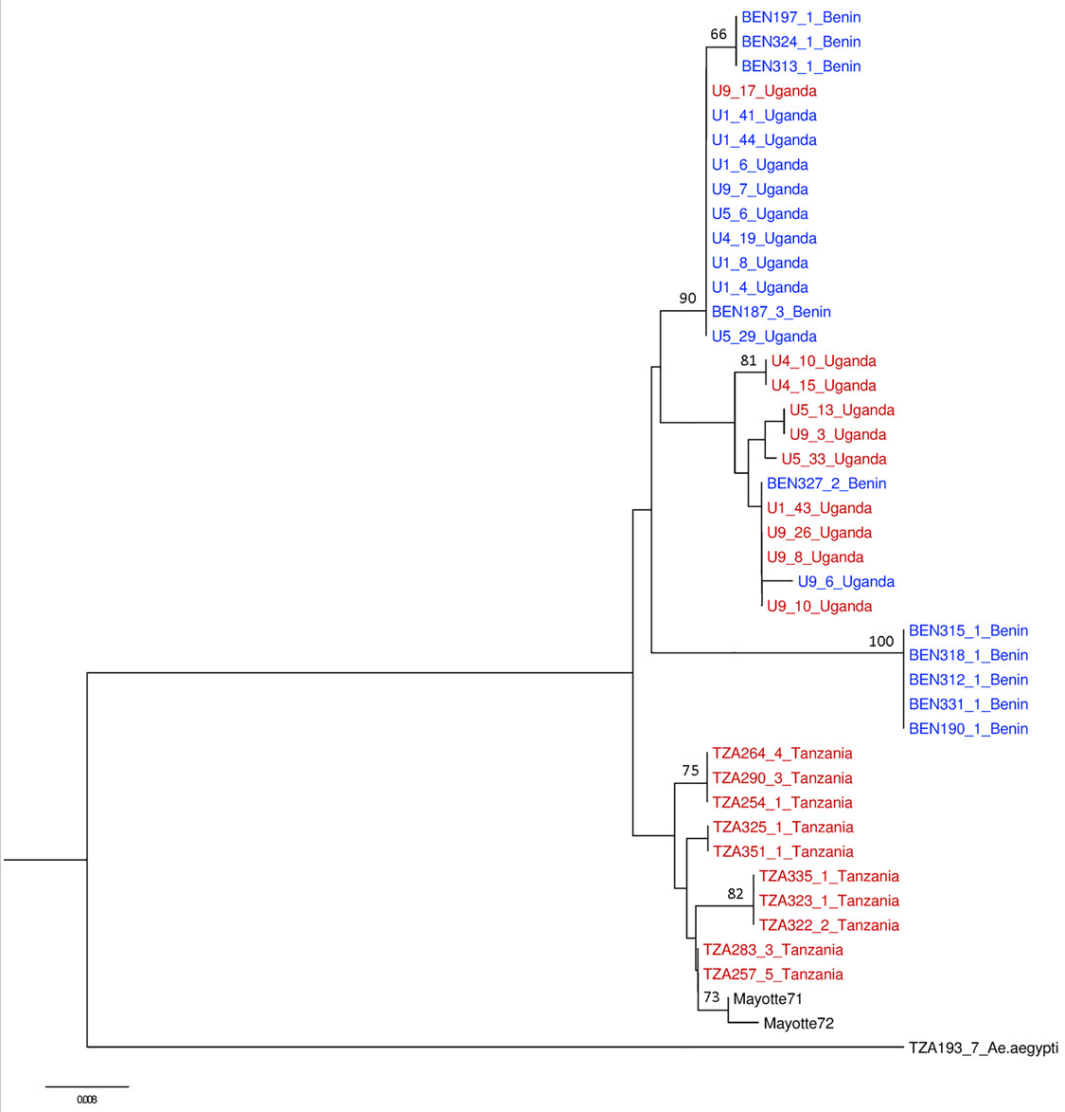
Neighbour joining tree of *COI* sequence data with labels coloured according to *ITS*-based species designation. Red labels represent *Ae*. *bromeliae* while blue labels are *Ae*. *lilii*. Sequences from Le Goff *et al*. [43] (^) are annotated. Bootstrap support values for branches above 55% are shown.

There are two phylogenetic clusters with high bootstrap support (90%, 90%) and two with weak bootstrap support (56% and 43%) in the NJ tree for mitochondrial *COI* with individuals tending to cluster according to geographic origin (Fig 5). The sequences from Mayotte cluster with *Ae*. *bromeliae* from Tanzania. Individuals designated as *Ae*. *bromeliae* or *Ae*. *lilii* according to *ITS* sequence variation tend to group according to species within the same clades, but there are three exceptions. One individual from Uganda, exhibiting the *ITS* sequence of *Ae*. *bromeliae* clusters within a grouping of *Ae*. *lilii* while two individuals from Uganda and Benin, identified as *Ae*. *lilii*, cluster within a grouping that is otherwise comprised of *Ae*. *bromeliae*. A hierarchical AMOVA showed there is more genetic variation within (42.83%) and between populations (55.18%) than between species (1.99%) at the *COI* gene.

### Species identification and geographical distribution

**Fig 6:**
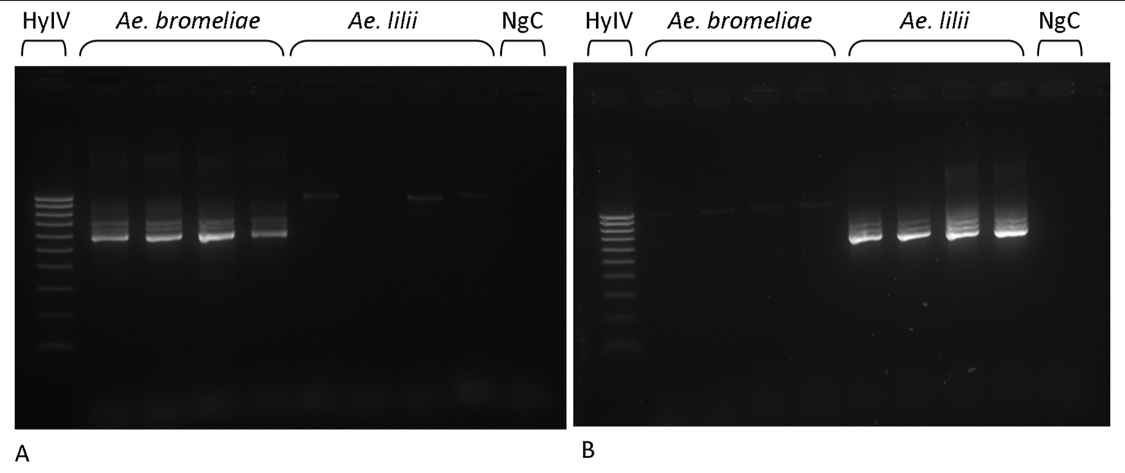
Electrophoresis gel for primer sets (A) BRO-F and BRO-R and (B) LIL-F and LIL-R. PCR products were run with Hyperladder IV (HyIV) and a negative control (NgC).

Based on fixed differences between species in the *ITS* sequences, species specific primers were designed to amplify PCR products in *Ae*. *bromeliae* or *Ae*. *lilii*. Primers designed to amplify in *Ae*. *bromeliae* did not amplify a PCR product from *Ae*. *lilii* and vice versa (Fig 6). Application of this method to 110 specimens of *Ae*. *simpsoni* s.l. positively identified all individuals as either *Ae*. *bromeliae* or *Ae*. *lilii*, as detailed in Table 1. Both species use the same breeding habitats in domestic and peridomestic habitats including the leaf axils of *Musa spp*., *Colocasia spp*. and *Dracena spp*. Despite focussed collection attempts, neither species was found utilising tree holes for immature development. Both species occur in sympatry in Uganda, while only *Ae*. *lilii* was collected from the Republic of Benin and *Ae*. *bromeliae* was the only species detected in Tanzania (Fig 1).

**Table 1:** The number of individuals (n = 110) identified from sampled locations using our PCR mediated identification method.

Country	Village/town		*Ae*. *bromeliae*	*Ae*. *lilli*
Tanzania	Mlimba		5	0
	Udagaji village		19	0
	Chita		1	0
	Mahenge		10	0
	Morningside		1	0
		**Total**	**36**	**0**
Benin	Pobe		0	14
	Niaouili Village		0	10
		**Total**	**0**	**24**
Uganda	Kanyawara		1	14
	Bundibugyo		14	6
	Kapchorwa		0	4
	Najjembe		5	6
		**Total**	**20**	**30**

## Discussion

Here we used multiple markers to confirm there are two closely related species of the Simpsoni Complex that occur both allopatrically and sympatrically across the sampled range in sub-Saharan Africa. These species correspond to *Ae*. *bromeliae* and *Ae*. *lilii* of the Simpsoni Complex as characterised by Mukwaya *et al*. [22, 27]. We infer that a third forest taxon originally reported as *Ae*. *lilii* in Walter *et al*. [28] relates to another species, possibly a another member of the Simpsoni Group. The development of a molecular species identification method for the YFV vector *Ae*. *bromeliae* and non-vector *Ae*. *lilii* using variation at the *ITS* region allowed us to make for the first time reliable inferences about mosquito ecology and distribution. Such information is vital for a complete understanding of disease transmission by these species.

All the *Ae*. *simpsoni* s.l. mosquitoes tested fell into one or other of two genetically divergent *ITS* lineages. Based on sequence similarity these correspond directly to the two lineages found by Mukwaya *et al*. [27] and we follow their previous designation as *Ae*. *bromeliae* and *Ae*. *lilii*. We consider their species designation to be reliable as it was based on both morphology and feeding preference. Firstly, even though some individuals could not be identified due to overlap in morphological characters, Mukwaya *et al*. [27] were able to identify some individual specimens from the defining characters of leg tarsomere banding pattern and claw morphology described by Huang [22, 23]. Secondly, Mukwaya *et al*. [27] identified species based upon host feeding behaviour; human landing catches were used to distinguish anthropophilic *Ae*. *bromeliae* from non-anthropophilic *Ae*. *lilii* which were collected as larvae where human biting members of the Simpsoni Complex were absent. We confirm the existence of *Ae*. *bromeliae* and *Ae*. *lilii* as two distinct species since, using *ITS* sequence variation, they remain genetically distinct in sympatry at several locations in Uganda.

Our study enabled us to link the work of Mukwaya *et al*. [27] with that of Walter *et al*. [28]. Whereas Muwakya *et al*. [27] focused on feeding behaviour, Walter *et al*. [28] found divergence between forest and peridomestic populations in coastal Kenya at three nuclear genes. Our phylogenetic analysis reveals that the genetic variation in the *ITS* region does not correspond to the two distinct lineages found in Walter *et al*. [28]. Both *Ae*. *bromeliae* and *Ae*. *lilii* fall into a single phylogenetic clade at nuclear genes *apoLp2* (apolipophorin 2) and *CYPJ92* (cytochrome p450). This is consistent with these more slowly evolving nuclear genes being unable to resolve recently diverged species due to incomplete lineage sorting [56]. The forest lineage from Kenya detected by nuclear genes *apoLp2* and *CYPJ92* is clearly distinct and has no shared ancestral polymorphism with either *Ae*. *bromeliae* or *Ae*. *lilii* indicating a more distant relationship. Morphological identification of mosquito species is notoriously difficult and there are many species within the wider Simpsoni Group which share morphological characteristics [26]. We therefore suggest that the forest species reported by Walter *et al*. [28] represents a member of the wider Simpsoni Group, rather than the Simpsoni Complex. Possible candidates for this species that are morphologically similar and known from Kenya include *Ae*. *gandaensis*, *Ae*. *woodi*, *Ae*. *subargenteus* and *Ae*. *sampi* [26].

The mitochondrial *COI* species barcode marker was also unable to distinguish these taxa with most genetic variation occurring between geographic populations rather than between species. Whilst this inability of *COI* to distinguish *Ae*. *bromeliae* and *Ae*. *lilii* could be due to incomplete lineage sorting, the phylogeny suggests it is most likely due to mtDNA introgression; there are three cases in which *Ae*. *bromeliae* clusters with *Ae*. *lilii* or vice versa, two of which occur in sympatry in Uganda. Mitochondrial introgression is fairly common between closely related mosquito taxa [33]. Given this, and that *Aedine* mosquitoes may be particularly prone to NUMT’s due to their large genome size [57] it would seem wise to not rely on mitochondrial markers to distinguish species in this genus without prior confirmation from an additional marker. We found that the *ITS* region was the only marker able to reliably separate species of the Simpsoni Complex and therefore used it to develop a PCR mediated species identification method. This method is widely applicable across the range of *Ae*. *bromeliae* and *Ae*. *lilii* except in southern Africa. For use in this region, the method should be modified to accommodate the presence of *Ae*. *simpsoni* s.s. Our PCR mediated species diagnostic method removes the difficulties imposed by morphological identification of field specimens and therefore provides a valuable asset to medical entomologists studying arbovirus transmission.

Our identification tool in conjunction with larval sampling from natural habitats has provided further information on the ecology of the Simpsoni Complex. Contrary to the wide range of plant species utilised by *Ae*. *bromeliae* as breeding habitats, the immatures of *Ae*. *lilii* have only been reported to date from the axils of *Sansevieria spp*., suggesting a narrower range of immature habitats [22, 23]. However, we found that *Ae*. *lilii*, like *Ae*. *bromeliae*, utilised the plant axils of *Musa spp*., *Colocasia spp*. and *Dracena spp*., suggesting that larval breeding sites are not as restricted as previously reported [22, 23]. Walter *et al*. [28] hypothesised that selective pressure for use of domestic larval habitats may have driven speciation in the Simpsoni Complex. This is not supported by our findings that showed no obvious differences in larval habitat between species, although our characterisation of larval habitats was not exhaustive. As host choice appears to be an ecological difference between these species, it is possible that divergent selection for anthropophily, and the reliable blood source it provides, could have driven species divergence [28, 58].

We have shown here that *Ae*. *bromeliae* and *Ae*. *lilii* are common in Uganda where they can be found in sympatry in peridomestic habitats. *Ae*. *lilii* was the only member of the Simpsoni Complex collected from the Republic of Benin where it is described for the first time, while only *Ae*. *bromeliae* was collected in Tanzania. In addition, Mukwaya *et al*.’s [27] sequence data shows that mosquitoes from Nigeria are *Ae*. *lilii* whereas those from Kenya are *Ae*. *bromeliae*. Molecular evidence therefore agrees with the earlier morphological data of Huang [22, 23] that *Ae*. *bromeliae* is prevalent in East Africa (Tanzania, Kenya, Uganda, Mayotte; Fig 1). This morphological data also indicates a wider distribution across sub-Saharan Africa for *Ae*. *bromeliae* (Fig 1) [22, 23], but it would be wise to confirm this using molecular identification. However, given that *Ae*. *bromeliae* tends to be readily collected when it is present, the molecular data suggest that only *Ae*. *lilii* is present in West Africa (Fig 1). This difference would be consistent with the early reports that West African populations are predominantly non-anthropophilic [59]. Based on our preliminary distribution data, *Ae*. *bromeliae* may be an important vector of yellow fever in East Africa where YFV has been isolated from the Simpsoni Complex previously and implemented in disease epidemics including the Ethiopian outbreak of 1960–61 [59–61]. In comparison, other mosquito vectors including *Ae*. *aegypti*, *Ae*. *luteocephalus*, *Ae*. *furcifer and Ae*. *taylori* may be more important for YFV disease transmission in West Africa where yellow fever has been isolated from these vectors and implicated in outbreaks [59, 62–65].

Emerging/re-emerging arboviruses such as Chikungunya and Zika are causing great concern since they are increasingly responsible for catastrophic epidemics worldwide [66–72]. Members of the Simpsoni Group transmit a range of arboviruses including yellow fever, Babanki and Ngari viruses [1]. A limited number of arboviral studies have focused on disease transmission in this species group, progress of which is hampered by incomplete taxonomic understanding. There is therefore a great need to resolve the molecular systematics of the wider Simpsoni Group that should in turn be used to develop methods of species identification. In addition to studies of arboviral risk, assessment of mosquito species ranges is required in order to relate these to differences in the distribution of arboviruses and/or arboviral genotypes such as that observed for yellow fever [59]. Improved identification methods would also facilitate studies on feeding behaviour and genetic introgression which are important for understanding the risks of zoonotic disease emergence. For example, the introgression we observed between *Ae*. *lilii* and *Ae*. *bromeliae* could increase the propensity of *Ae*. *lilii* to feed on humans, resulting in the increased transfer of zoonotic disease into humans. A precedent for this can be seen in *Culex pipiens* in North America where mixing of the molestus and pipiens genetic forms (predominantly human and bird feeding, respectively) increases transmission of West Nile virus to humans. A wide range of inferences on ecology and epidemiology can now be made with our molecular identification tool that should be used to assess disease risk and provide basic information for vector population control.

## Supporting Information

S1 Table Sampling information for Simpsoni Complex mosquitoes and the number of individuals sequenced at nuclear and mitochondrial genes. (XLSX)
